# Exploration of the Structure–Function Relationships of a Novel Frog Skin Secretion-Derived Bioactive Peptide, t-DPH1, through Use of Rational Design, Cationicity Enhancement and In Vitro Studies

**DOI:** 10.3390/antibiotics10121529

**Published:** 2021-12-14

**Authors:** Haixin Qin, Hantian Fang, Xiaoling Chen, Lei Wang, Chengbang Ma, Xinping Xi, Tianbao Chen, Chris Shaw, Mei Zhou

**Affiliations:** School of Pharmacy, Queen’s University Belfast, 97 Lisburn Road, Belfast BT9 7BL, UK; hqin03@qub.ac.uk (H.Q.); hfang02@qub.ac.uk (H.F.); l.wang@qub.ac.uk (L.W.); c.ma@qub.ac.uk (C.M.); x.xi@qub.ac.uk (X.X.); t.chen@qub.ac.uk (T.C.); chris.shaw@qub.ac.uk (C.S.)

**Keywords:** antimicrobial peptides, frog skin secretion, dermaseptin, peptide modification

## Abstract

Amphibian skin-derived antimicrobial peptides (AMPs) have attracted increasing attention from scientists because of their excellent bioactivity and low drug resistance. In addition to being the alternative choice of antibiotics or anticancer agents, natural AMPs can also be modified as templates to optimise their bioactivities further. Here, a novel dermaseptin peptide, t-DPH1, with extensive antimicrobial activity and antiproliferative activity, was isolated from the skin secretion of *Phyllomedusa hypochondrialis* through ‘shotgun’ cloning. A series of cationicity-enhanced analogues of t-DPH1 were designed to further improve its bioactivities and explore the charge threshold of enhancing the bioactivity of t-DPH1. The present data suggest that improving the net charge can enhance the bioactivities to some extent. However, when the charge exceeds a specific limit, the bioactivities decrease or remain the same. When the net charge achieves the limit, improving the hydrophobicity makes no sense to enhance bioactivity. For t-DPH1, the upper limit of the net charge was +7. All the designed cationicity-enhanced analogues produced no drug resistance in the Gram-negative bacterium, *Escherichia coli*. These findings provide creative insights into the role of natural drug discovery in providing templates for structural modification for activity enhancement.

## 1. Introduction

Natural antimicrobial peptides (AMPs), as a critical component in the fight against infectious pathogens [1], are considered to be the ideal alternative agents to traditional antibiotics due to the fact of their broad-spectrum antimicrobial activity and low potential for inducing drug resistance [2,3]. Furthermore, in playing an essential role in their immune systems [4], the skin secretions of amphibians are known as effective sources for AMPs [5,6].

Despite their diversity, AMPs have characteristic features, including conformation, net charge, hydrophobicity and amphipathicity, all of which are associated with their ability to kill microorganisms [7]. The membrane permeability caused by these factors is a widely accepted mechanism of AMP actions [8,9]. Briefly, the cationic AMP molecules initially associate with the anionic phospholipids and acidic polymers of the cell membrane through electrostatic interaction [10]. Then, these AMPs adopt amphiphilic secondary α-helix conformations. The hydrophobicity of AMPs promotes the interaction between peptides and fatty acyl chains to form pores or align parallel to the surface on the cell membrane and to disrupt the cell membrane [11,12,13]. Thus, it can be seen that the interaction with AMPs and the anionic surface of the membrane is a prerequisite for their membrane permeabilising actions [14]. On this basis, Du et al. constructed two cationicity-enhanced analogues of novel AMPs, AcrAP1 and AcrAP2, by using positively charged lysine residues to substitute the neutrally charged serine residues. The modified products exhibited a more potent antimicrobial activity in killing *Staphylococcus aureus* (*S. aureus*), *Escherichia coli* (*E. coli*) and *Candida albicans* (*C. albicans*) [15]. In addition, Chen et al. modified the natural peptide PAM-37 by replacing phenylalanine at position 34 with positively charged arginine. The modified peptide PAM-37 (F34-R) obtained one more net charge and displayed better antimicrobial activity against *S. aureus*, *Listeria monocytogenes*, *Salmonella typhimurium* and *Pseudomonas aeruginosa* (*P. aeruginosa*) [16]. In addition, some researchers tried to replace the cationic amino acids in AMPs, which mostly resulted in a significant decrease or complete loss of antimicrobial effect, indicating the high importance of cationic residues [17,18]. The natural peptide Thanatin, for example, showed excellent antimicrobial activity against a variety of pathogens including drug-resistance; however, when the positively charged R13 and R14 were replaced by alanine, the recognition of the outer membrane lipopolysaccharide was affected, resulting in the loss of antimicrobial activity [19]. That is to say, when taking novel peptides as templates for modification, increasing net charge is a feasible starting point to increase their bioactivity.

Dermaseptin peptides form a highly conserved family with the typical amphiphilic consensus motif (AAXKAALXK, X can be any amino acid), a conserved tryptophan residue at the third position and C-terminal amidation [20]. Thus far, more than 100 peptides have been proved to belong to the dermaseptin family. Dermaseptin PS1 showed significant proliferative inhibition on more than 16 human cancer cell lines [21]. In addition, dermaseptin S4 and its analogues were effective against nine reference and clinical *Neisseria gonorrhoeae* strains [22]. Dermaseptin PS4 displayed better inhibition of cell proliferation than melittin when acting against the U251MG, H157 and MDA-MB-435S cell lines [23]. The amide group and positively charged amino acid provide the cationic of dermaseptins, and the consensus motif provides the amphiphilic domain [24]. These are the structural bases for the excellent bioactivity of dermaseptin family peptides. Meanwhile, novel dermaseptins have great potential to be used as peptide modification templates to improve their bioactivity further.

Here, we identified a novel dermaseptin peptide named t-DPH1 from *Phyllomedusa hypochondrialis* (*P. hypochondrialis*) through shotgun cloning. A series of bioactivity assessments demonstrated that this peptide had broad antimicrobial activities and potential antiproliferative activity with a mechanism of action through destroying the cell membrane. In addition, four cationic-enhanced analogues, with net charges ranging from +5 to +7, were designed to optimise the bioactivity of t-DPH1 and to further investigate the net charge threshold on the bioactivity of the peptide.

## 2. Results

### 2.1. Molecular Cloning of cDNA Encoding t-DPH1 Precursor

**Figure 1:**
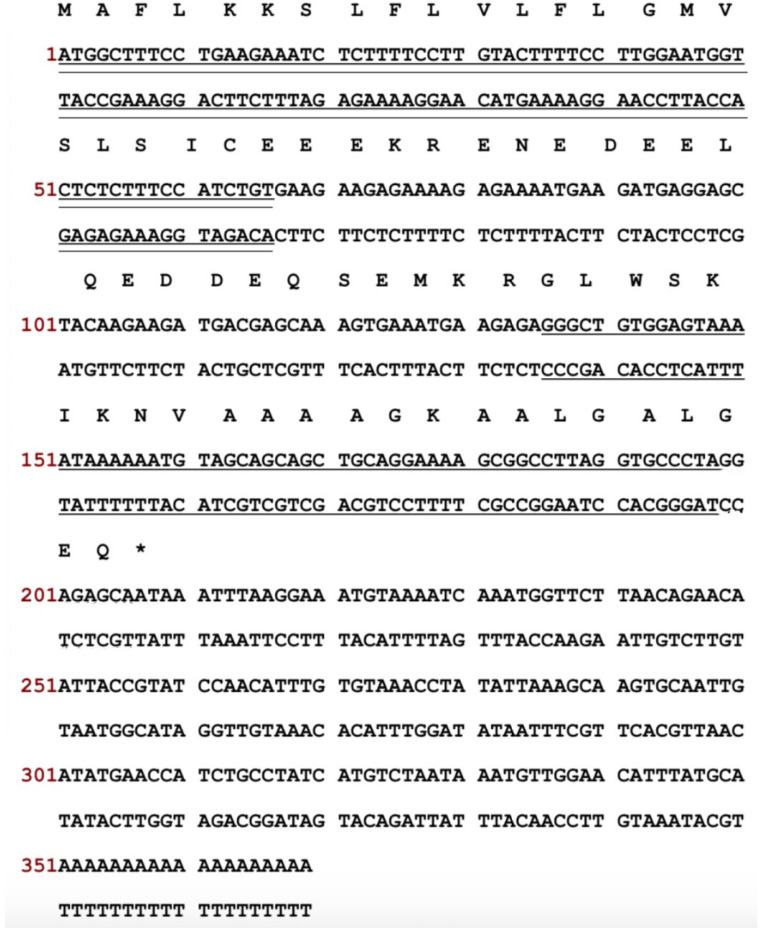
The nucleotides and translated open reading frame of the cloned cDNA from the skin secretion of *P*. *hypochondrialis*. The putative signal peptide is marked with double-underline. The mature peptide is marked with a single underline, and the stop codon is labelled by an asterisk.

**Figure 2:**
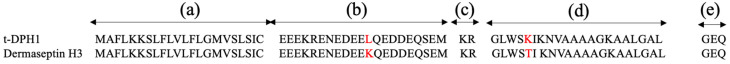
Multiple alignments of precursors encoding t-DPH1 and dermaseptin H3: (**a**) signal peptide; (**b**) acidic spacer peptide region; (**c**) enzyme cleavage site; (**d**) mature peptide; (**e**) glycine residue amide donor. The different amino acids between the two sequences are highlighted in red.

The cDNA encoding the peptide precursor of a novel peptide was repeatedly cloned from a skin secretion cDNA library of *P. hypochondrialis* (Figure 1). The translated open reading frame was made up of 69 amino acid residues including a putative signal peptide domain (22 residues), an acidic peptide spacer (21 residues), a typical-KR-pro-peptide cleavage site and a mature peptide (21 residues). An extension-GEQ-sequence was also present in which the glycine residue served as the donor of C-terminal amidation of the mature peptide. The novel peptide was named t-dermaseptin-PH1 (t-DPH1) according to its origin as suggested by nomenclature from the APD3 antimicrobial peptide database (https://aps.unmc.edu/nomenclature, accessed on 3 December 2021). The prefix ‘t’ and the trailing number ‘1’ were used to distinguish it from published peptides. Compared with a reported dermaseptin peptide, the sequences of t-DPH1 and dermaseptin H3 shared a highly conserved domain at over 90% (Figure 2). Thus, t-DPH1 was confirmed as a novel member of the dermaseptin peptide family. The cDNA sequence of the t-DPH1 precursor has been deposited in the NCBI Database under an accession number: OK631446.

### 2.2. Purification and Identification of Synthesised t-DPH1 and Its Analogues

After synthesis, the novel peptide t-DPH1 and its analogues were purified by reverse-phase HPLC. The relevant chromatographs are shown in Supplementary Materials Figure S1. Then, MALDI-TOF mass spectrometry (Figure S2) confirmed the molecular mass of each peptide.

### 2.3. Peptide Design and Secondary Structure Prediction Analysis of t-DPH1 and Its Analogues

The sequence of the natural peptide (t-DPH1) was established as GLWSKIKNVAAAAGKAALGAL-NH_2_, and it was used as a template to design four analogues: t-DPH1-K4, t-DPH1-5K, t-DPH1-6K and t-DPH1-6KW, which aimed to explore the structure–function relationship and to enhance the antimicrobial activity and antiproliferative activity of the parent peptide. The main idea of modification was to increase the net charge by amino acids substitution based on the cationic property of the parent peptide. The charge threshold for enhancing the bioactivity of the parent peptide was investigated by observing the changes in the bioactivity of modified analogues. First, the serine residue at the 4th position of t-DPH1 was replaced by a positively charged lysine residue to construct a new analogue termed t-DPH1-K4 with one more net charge (+5) than t-DPH1. In addition, lysine residues were used to replace the residues at positions 4 and 11 of t-DPH1 to design t-DPH1-5K, with two more net charges (+6) than t-DPH1. Then, the third analogue with a net charge of +7, t-DPH1-6K, was designed using three lysine residues to replace the residues S4, A11 and L18 of t-DPH1. Finally, another analogue, t-DPH1-6KW, was constructed based on t-DPH1-6K. The difference was that the residue at the first position changed to tryptophan, which achieved higher amphipathicity than that of t-DPH1-6K.

**Figure 3:**
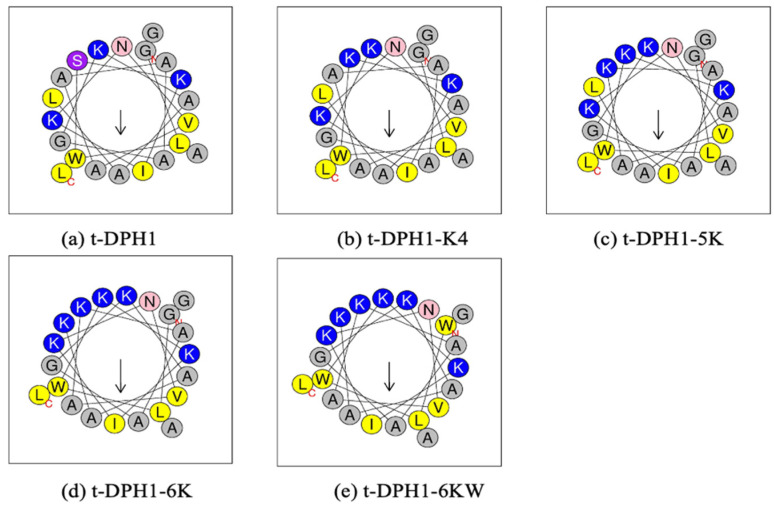
Predicted helical wheel plots of t-DPH1 and its analogues (**a**–**e**). The arrow in each picture points to the hydrophobic face of the peptide. Amino acids with different properties are presented in different colours (grey: non-polar residue; blue: positively charged residue; yellow: hydrophobic residue, pink: asparagine; purple: serine).

The helical wheel plots and physiochemical properties of t-DPH1 and its designed analogues were analysed by the online analysis tool Heliquest. The novel peptide t-DPH1 possessed a +4 net charge with a hydrophobicity of 0.425 and a hydrophobic moment of 0.337 (Table 1). All the synthetic peptides had the same hydrophobic face (AVLAAIAAWL) (Figure 3). When designing t-DPH1-K4, t-DPH1-5K and t-DPH1-6K, positively charged lysine residues were chosen to replace one or more amino acids located at the hydrophilic face to construct a series of cationic-enhanced analogues. These analogues also achieved better amphipathicity, from 0.414 to 0.513. As for t-DPH1-6KW, G^1^ was replaced by the hydrophobic tryptophan residue, resulting in increased hydrophobicity (0.297) while maintaining the same net charge as t-DPH1-6K (+7).

**Figure 4:**
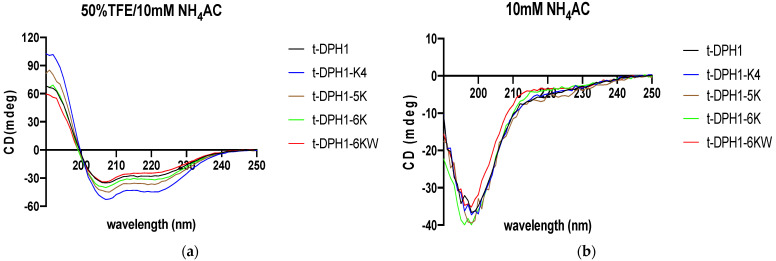
Circular dichroism spectra of t-DPH1 and its analogues (100 μM) in 50% TFE/NH_4_AC (**a**) and 10 mM NH_4_AC buffer (**b**).

In an aqueous solution (10 mM NH_4_Ac), all tested peptides displayed random coil structures but tended to fold into a helical structure in a membrane-mimetic environment (50% TFE in 10 mM NH_4_Ac solution) (Figure 4). The proportion of % of α-helix structure domains in 50% TFE were calculated using the online tool K2D3 webserver (Table 2). All the modified peptides demonstrated a higher proportion of α-helix structure than the parent peptide t-DPH1. Among these, t-DPH1-K4 with a net charge of +5 showed the highest proportion of α-helix structure (69.33%).

### 2.4. Antimicrobial Activities of t-DPH1 and Its Analogues

The values for the minimum inhibitory concentrations (MICs) and minimum bactericidal concentrations (MBCs) of t-DPH1 and its analogues are shown in Table 3. The parent peptide t-DPH1 showed broad-spectrum antimicrobial activity against all the tested seven microorganisms, especially in killing Gram-negative *E. coli* with an MIC of 2 μM and MBC of 4 μM. As for the enhanced cationic analogues, t-DPH1-K4 and t-DPH1-5K, with net charges of +5 and +6, displayed an even stronger effect against all the tested microorganisms. Moreover, t-DPH1-6K retained similar antimicrobial activity against *E. coli* and achieved a better effect in killing *Klebsiella pneumoniae* (*K. pneumoniae*), *P. aeruginosa* and *S. aureus*. However, t-DPH1-6KW, with the same net charge as t-DPH1-6K (+7), decreased in potency against all the tested microorganisms except for *E. coli.*

### 2.5. Anti-Biofilm Activities of t-DPH1 and Its Analogues

The values of the minimum biofilm inhibitory concentrations (MBICs) and minimum biofilm eradication concentrations (MBECs) of t-DPH1 and its analogues are shown in Table 4. Except for t-DPH1-6KW, all the synthetic peptides were capable of inhibiting the formation biofilm of tested bacteria but did not have obvious eradication activities on the formed biofilms. Among all the tested peptides, t-DPH1-5K seemed to be the most potent analogue for antibiofilm activity.

### 2.6. Time–Killing Kinetics of t-DPH1, t-DPH1-K4, t-DPH1-5K and t-DPH1-6K

**Figure 5:**
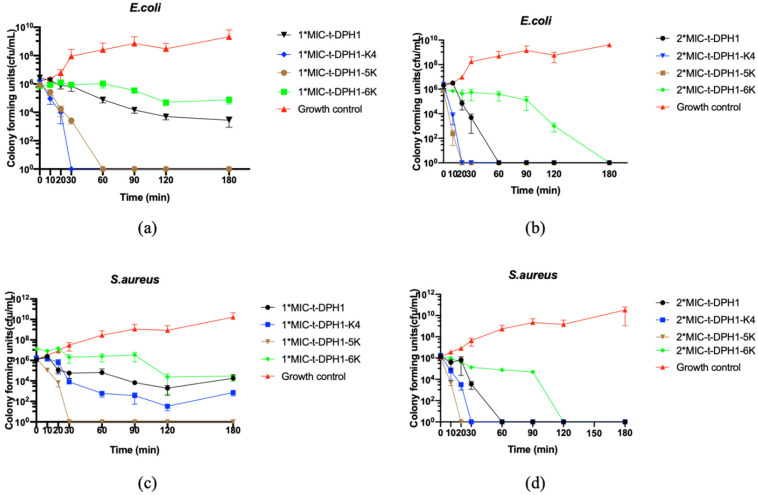
The time–killing curves of t-DPH1, t-DPH1-K4, t-DPH1-5K and t-DPH1-6K against *E. coli* and *S. aureus* over 180 min. (**a**) Peptides against *E. coli* at the concentrations of 1*MIC; (**b**) Peptides against *E. coli* at the concentrations of 2*MIC; (**c**) Peptides against *S. aureus* at the concentrations of 1*MIC; (**d**) Peptides against *S. aureus* at the concentrations of 2*MIC. The error bars represent the mean ± SD of nine replicates.

The time–killing curves demonstrated that t-DPH1, t-DPH1-K4, t-DPH1-5K and t-DPH1-6K were able to kill *E. coli* and *S. aureus* at their corresponding two-fold MICs in 180 min (Figure 5). At the corresponding MICs, only t-DPH1-5K was able to kill *S. aureus*. Meanwhile, t-DPH1-K4 showed the highest efficiency in killing *E. coli* at MIC. It could kill the bacteria in 30 min, a much shorter time than that of t-DPH1-5K. In terms of the number of colony formations under MICs, all the tested peptides could cause a significant reduction in the number of colonies of *E. coli* and *S. aureus* in 180 min. Still, the inhibitory effect of t-DPH1-6K was weaker than that of the parent peptide t-DPH1.

### 2.7. Permeabilisation Effects of t-DPH1 and Its Analogues on the Bacterial Cell Membrane

**Figure 6:**
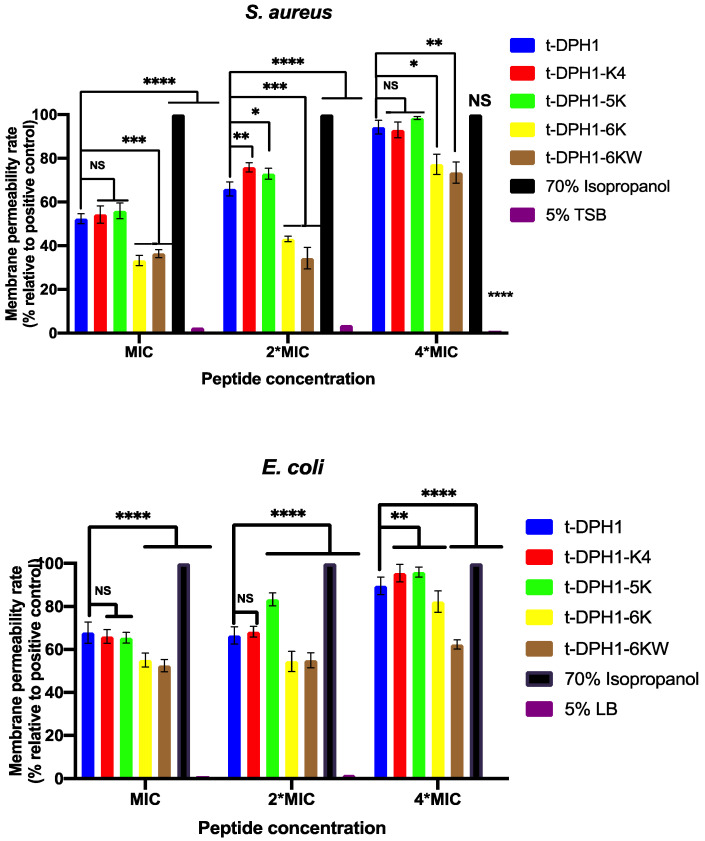
The destruction of *S. aureus* and *E. coli* cell membranes at concentrations of MIC, 2*MIC and 4*MIC. Bacteria treated with 70% isopropanol and 5% TSB or LB served as the positive control and negative control groups. The data were analysed by multiple *t*-tests comparing the membrane permeability rate of different peptides and control groups with that of t-DPH1 in the same concentration, and the significance is indicated by asterisks (* *p* < 0.05; ** *p* < 0.01; *** *p* < 0.001; **** *p* < 0.0001) and NS represented no significant difference. The error bar represents the SD of nine replicates.

As shown in Figure 6, t-DPH1 and its analogues could cause permeabilisation of the cell membrane of both *S. aureus* and *E. coli* in two hours. Except for t-DPH1-6KW, the permeability rates of t-DPH1 and its analogues were positively correlated to concentrations. Among them, t-DPH1-K4 and t-DPH1-5K demonstrated a similar permeability rate at the corresponding MIC. When the concentrations increased to their corresponding 2*MIC, the membrane permeability rate of t-DPH1-5K against the two bacteria increased compared with the parent peptide. Nevertheless, the permeabilisation effect of t-DPH1-K4 on the cell membrane of *E. coli* was still similar to that of t-DPH1. When the concentration increased to their corresponding 4*MIC, the permeability rate of these two peptides on the cell membrane of *E. coli* was more than 80%. The other two analogues, t-DPH1-6K and t-DPH1-6KW, showed a weaker cell membrane permeability effect than that of the parent peptide at all tested concentrations.

### 2.8. Assessments of Resistance Induction of t-DPH1 and Its Analogues

**Figure 7:**
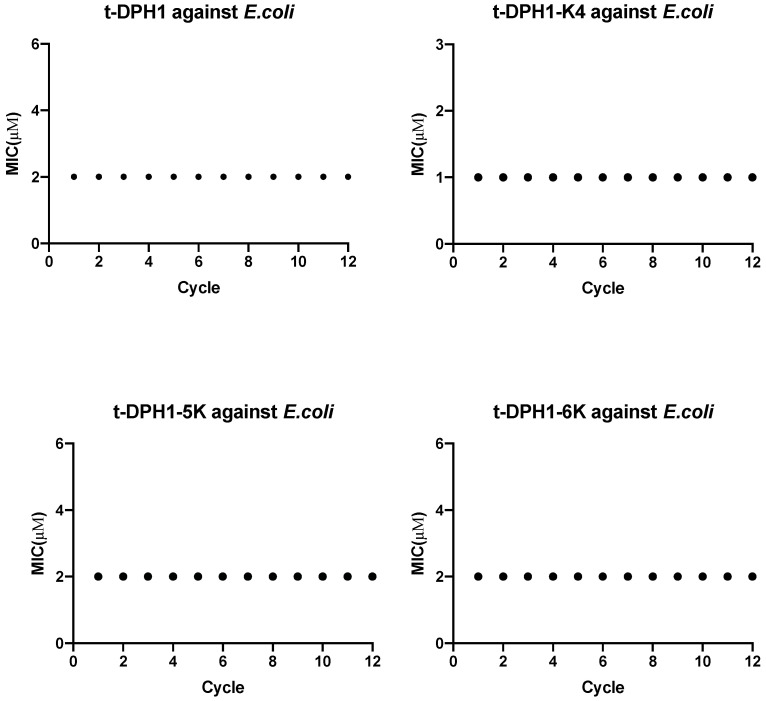
Assessment of resistance induction of t-DPH1, t-DPH1-K4, t-DPH1-5K and t-DPH1-6K against *E. coli* over 12 passages.

The serial passages of Gram-negative *E. coli* treated with t-DPH1, t-DPH1-K4, t-DPH1-5K and t-DPH1-6K did not increase specific bacterial resistance. The corresponding MICs of these peptides against *E. coli* were consistent over 12 cycles (Figure 7).

### 2.9. Anti-Proliferation Activities of t-DPH1 and Its Analogues

**Figure 8:**
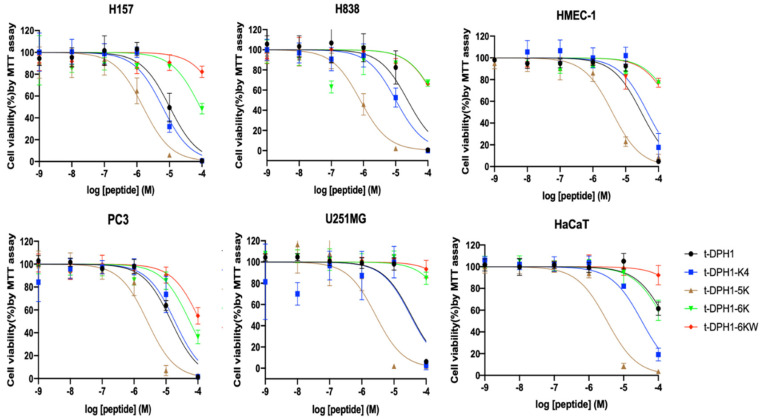
Viability of four cancer cell lines (H157, H838, PC3 and U251MG) and two normal human cell lines (HMEC-1 and HaCaT) after treatment with t-DPH1 and its analogues for 24 h. The error bar indicated the SD of nine replications.

The novel peptide t-DPH1 displayed a broad-spectrum antiproliferative effect against the PC-3, H838, H157 and U251MG cancer cell lines, with IC_50_ values from 10.20 to 34.25 μM, but it also showed an inhibitory effect towards the proliferation of normal human cell lines HMEC-1 (IC_50_: 29.85 μM) and HaCaT (175.6 μM) (Table 5). The enhanced cationic analogues, t-DPH1-K4 and t-DPH1-5K, showed enhanced potency in killing the tested cancer cell lines, especially human non-small lung cancer cell lines H838 and H157. Meanwhile, t-DPH1-K4 had a lower inhibitory effect on the proliferation of HMEC-1 than t-DPH1. It was unexcepted that t-DPH1-6K and t-DPH1-6KW, with three more net charges than t-DPH1, were less effective in inhibiting the growth of the tested cancer cell lines based on the current observation. However, even at the high concentration (100 μM), they slightly influenced the growth of the tested cancer cell lines (Figure 8).

### 2.10. Haemolysis Activities of t-DPH1 and Its Analogues

**Figure 9:**
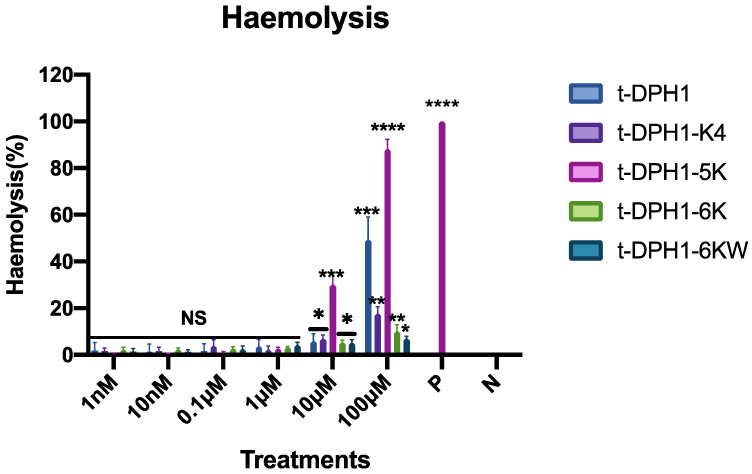
Haemolytic activities of t-DPH1 and its analogues against horse red blood cells. ‘P’ represents the positive control group, and ‘N’ represents the negative control group, 0.1% Triton X-100 and PBS, respectively. The data were analysed by two-way ANOVA by comparing the haemolysis rates of different peptides in a series of concentrations with the negative control group. The significance is demonstrated with asterisks (* *p* < 0.05; ** *p* < 0.01; *** *p* < 0.001; **** *p* < 0.0001), and NS means there is no significant difference. The error bar indicates the SD of nine replicates.

As shown in Figure 9, the parent peptide t-DPH1 displayed over 50% haemolysis at a high concentration (100 μM). Except for t-DPH1-5K, all the other designed analogues of t-DPH1 possessed lower haemolytic activity at the tested concentrations (Table 6). Despite the enhanced antimicrobial activities, t-DPH1-K4 and t-DPH1-6K had lower haemolytic activities. The values of HC_50_ were four and eight times larger than that of t-DPH1, respectively. But the haemolytic activity of t-DPH1-5K was nearly five times higher than that of t-DPH1. Among all the designed analogues, t-DPH1-6KW demonstrated the lowest haemolytic activity. It almost did not show any haemolytic activity at the test concentration.

## 3. Discussion

Amphibian skin-derived peptides have been closely studied for over 50 years, many of which have been proved to have considerable bioactivities including working as potential antimicrobial agents, protease inhibitors, smooth muscle relaxants, insulin-releasing promoters, and others [23,24,25,26,27]. In addition, natural AMPs can serve as templates to be modified by single or multi-specific site amino acid substitution to achieve higher molecular diversity and enhanced properties [15]. That was the strategy adopted in this study.

The novel peptide t-DPH1, first discovered in this study, is a linear cationic AMP belonging to the dermaseptin family. The extensive antimicrobial activity of t-DPH1 was demonstrated against the tested seven microorganisms. Although Gram-negative bacteria have a thicker cell wall (composed of lipopolysaccharide) than those of Gram-positive bacteria (composed of peptidoglycan) [28], t-DPH1 still exhibited more potent abilities against Gram-negative bacteria. Meanwhile, t-DPH1 demonstrated an inhibitory effect on the growth of four tested human cancer cell lines. As previously stated [29], cationicity is the initial factor in interacting AMPs with cell membranes. Thus, when modifying parent peptide t-DPH1, positively charged lysine residues were used to substitute the amino acids located at the hydrophilic face of the sequence of t-DPH1, and a series of analogues with enhanced cationicity were designed.

The secondary structure and physicochemical properties of t-DPH1 and its analogues were analysed by online bioinformatic tools, and the CD spectra confirmed that, like most AMPs (e.g., magainin 2, temporin), t-DPH1 and its analogues tended to form random coils in an aqueous environment while forming a stable alpha-helix structure in a membrane environment [30,31]. This amphipathic conformation is a vital factor needed by AMPs for their bioactivities [32]. Therefore, based on the predicted helical wheel plots, replacing one or more amino acids with a hydrophilic positively charged lysine residue on the hydrophilic surface not only improved the net charge of the parent peptide but enhanced the amphiphilicity as well. Accordingly, the α-helix degree of all modified peptides was improved compared with that of the parent peptide.

The bioactivity of the analogues was observed and compared with that of the parent peptide, and it was found that t-DPH1-K4 and t-DPH1-5K, with net charges of +5 and +6, achieved overall promotion in antimicrobial activity, especially in killing Gram-negative bacteria. Furthermore, compared with the parent peptide, t-DPH1-K4 and t-DPH1-5K had higher net charges and stronger amphiphilicity, which made it easier for them to approach and penetrate negatively charged lipopolysaccharides in the outer membrane of Gram-negative bacteria and interact with relatively weak peptidoglycans in the cell wall, leading to cell rupture [33]. As a previous study proved, the anti-biofilm effect of cationic AMPs can be exerted through the mechanisms of interacting with the cell membrane, including disrupting the membrane potential of cells embedded by degrading the biofilm matrix and polysaccharides, etc. [34]. In the assessment of anti-biofilm activity, t-DPH1-K4 and t-DPH1-5K also showed an eradication effect on the formed Gram-negative bacterial biofilm, which was not observed in other tested peptides. These confirmed the contribution of the positively charged amino group on the side chain of lysine, enhancing the cationic peptide’s binding to the negatively charged cell membrane [35].

The bacteria cell membrane permeability assay and time–killing assay showed that t-DPH1 and its analogues had concentration-dependent damage to bacterial cell membranes. This may be because peptides can only form a limited number of pores in the cell membrane at low concentrations. High concentrations of peptides tend to accumulate on the membrane, resulting in membrane rupture [36]. At the same concentration (their corresponding 2*MICs), there was no significant difference in the membrane permeability rate of t-DPH1-K4 to *E. coli* cell membrane compared with that of t-DPH1. But t-DPH1-K4 could kill *E. coli* much faster than t-DPH1 at their corresponding 2*MICs. Similarly, at the MIC concentration, the membrane permeability rate of *S. aureus* cell membrane caused by t-DPH1-5K was similar to that of t-DPH1 at their corresponding MICs, but it could kill bacteria within 30 min, which was much faster than that of t-DPH1. These phenomena indicate that increasing the net charge can make AMPs approach the cell membrane faster and, thus, quicken the bactericidal speed, but it cannot further improve the permeability effect of the peptide.

In addition to excellent antimicrobial activity, t-DPH1-K4 and t-DPH1-5K also had a stronger anti-proliferation effect on the tested non-small lung cancer cells than that of the parent peptide. Compared with normal cells, there are more anionic molecules on the surface of cancer cells such as O-glycosylated mucins, heparin sulphate, and sialylated gangliosides [37]. The enhanced cationic properties promoted the interaction between peptides and cancer membranes. However, although t-DPH1-5K can kill cancer cells at low concentrations, it can also inhibit normal cell proliferation, limiting its further application. The cytotoxicity of t-DPH1-K4 to normal cells was higher than that of the parent peptide, but it was still in an acceptable range.

However, when the net charge was further improved to +7, the antimicrobial activity of t-DPH1-6K was not significantly improved compared with that of t-DPH1-5K, but the antiproliferative activity decreased dramatically. Keeping the same quantity of net charge, the hydrophobicity was adjusted by replacing the Glycine^1^ residue with the hydrophobic tryptophan residue. Still, the product t-DPH1-6KW showed no other ideal bioactivity except the bactericidal activity against Gram-negative *E. coli*. Given this result, it is necessary to discuss the relationship between the activity of AMPs and the range of net charge. Some studies believe that the charge range of AMPs with excellent activity is generally between +3 and +6 [38]. However, the optimal amount has not been decided yet, which may also be related to the length of the peptide sequence, secondary structure and amino acid types of different peptides [39]. A recent report showed that DFT503, an AMP containing one lysine, performed favourable activities both in in vitro and in vivo studies against antibiotic-resistant Gram-positive bacteria; however, DFT564 and DFT565, which contained three and four lysine residues, lost their antibacterial potency when additional basic amino acids were added, indicating that high cationic property was the main cause of its functional failure [40]. On the one hand, when the net charge is higher than the upper limit, the bioactivities of AMPs may be reduced due to the mutual repulsion of the same charges so that the peptide could not bind with the cell membrane [41]. On the other hand, the excessive hydrophilic residues may reduce the hydrophobicity of AMPs which then cannot be deeply inserted into the cell membrane, thus being unable to significantly improve the effect [32]. These theories might explain the failure of t-DPH1-6K and t-DPH1-6KW. The balance between charge quantities and other properties, including hydrophobicity and amphiphilicity, is important in peptide modification. When the charge of AMPs reaches the threshold value, although the amphiphilicity and hydrophobicity of t-DPH1-6K and t-DPH1-6KW were within a reasonable range, their bioactivities were still affected by the excessive net charge.

It is worth mentioning that all the tested peptides kept consistent MICs against *E. coli* in a resistance induction experiment over 12 cycles. At present, the drug resistance of *E. coli* poses a serious threat to human health. The results showed that the resistance of *E. coli* to ciprofloxacin, gentamicin, trimethoprime/sulfamethoxazole and third-generation cephalosporin increased significantly with the duration of drug administration [42]. For the conventional antibiotic, ampicillin, resistance has increased to 50% or higher in high-risk populations [43]. Therefore, when evaluating the antibacterial effect of new antibiotics, the characteristics of drug resistance should be taken into consideration. Although we cannot rely on these data to speculate the long-term effect, it does prove an advantage compared to traditional antibiotics. There is a low possibility for AMPs to induce drug resistance [11], which is due to the mechanism of AMPs in that they bind with the cell membrane on multi-targets which disrupts the cell membrane rapidly [9]. Furthermore, the designed analogues t-DPH1-K4 and t-DPH1-5K could kill the bacteria in 30 min at their corresponding MBCs, whereas the traditional antibiotics may take 6 to 12 h or more to achieve the same effect [44].

In addition, to develop AMPs into alternative antibiotics and anticancer agents, haemolytic activity is an important index that must be paid attention to. Although t-DPH1-5K had the strongest antimicrobial and antiproliferative activities, it produced a nearly 40% haemolysis at the concentration of 10 μM. This may be due to the high amphiphilicity of the peptide, which induced a strong hydrophobic interaction with the cell membrane [45]. The wide range of bioactivity makes t-DPH1-5K a great choice of antimicrobial drug lead, but it still needs further modification to decrease its haemolytic activity. By contrast, t-DPH1-K4 showed a wider therapeutic window and had the potential to become one of the potential drug candidates with great antimicrobial and anticancer dual effects, just like cecropins and magainins [46].

Last but not least, this study used a lysine substitution strategy to enhance the cationicity of the parent peptide t-DPH1. However, in recent years, many studies have shown that the guanidinium moiety of arginine, which is also positively charged, has a stronger H-bonding capability and is more effective in mediating the peptide-membrane interaction [47,48,49]. Thus, this may be one of the ideas to improve the bioactivities of t-DPH1-K4 and t-DPH1-5K further.

## 4. Materials and Methods

### 4.1. Skin Secretion Harvesting from P. hypochondrialis

The dorsal skin secretions of adult specimens of *P. hypochondrialis* were obtained by electrical stimulation (5 V, 100 Hz, 140 ms width) [50] through platinum electrodes for every 20 s. The skin secretions were rinsed off the skin with deionised water into a chilled beaker and stored at −20 °C, then snap-frozen in liquid nitrogen and lyophilised. The study was performed according to the guidelines in the UK Animal (Scientific Procedures) Act 1986, project license PPL 2694, issued by the Department of Health, Social Services and Public Safety, Northern Ireland. Procedures were vetted by the Institutional Animal Care and Use Committee (IACUC) of Queen’s University Belfast and approved on 1 March 2011.

### 4.2. Identification of Precursor-Encoding cDNAs from the Skin Secretion

Poly-A mRNA was extracted by Dynabeads mRNA Direct kit (Dynal Biotech, Wirral, UK) due to covalent pairing and was made into a first-strand cDNA library. Then a SMART-RACE Kit (Clontech, Oxford, UK) and a degenerate sense primer (S1; 5’-ACTTTCYGAWTTRYAAGMCCAAABATG-3’ (Y = C/T, W = A/T, R = A/G, M = A/C, B = T/C/G) were used to conduct the process of RACE-PCR so that full-length sequences of the mRNA could be obtained. The PCR products were subjected to purify and cloned by using a pGEM^®^-T Easy Vector system (Promega, Southampton, UK) and sequenced by an ABI 3100 Automated Capillary Sequencer (Applied Biosystems, Forster City, CA, USA). Online APD3 antimicrobial peptide database (https://aps.unmc.edu, accessed on 3 December 2021) was used to find out the sequence similarity between t-DPH1 and other reported AMPs.

### 4.3. Peptide Synthesis

Parent peptide t-DPH1 and its designed analogues were synthesised using a Tribute peptide synthesiser (Protein Technologies, Tucson, AZ, USA). The products were purified by reverse-phase HPLC (Phenomenex Aeris PEPTIDE 5 µm XB-C18 column, 250 × 21.2 mm, Macclesfield, Cheshire, UK) with a linear gradient formed from 80% buffer A (0.05/99.5 (*v*/*v*) TFA/water) and 20% buffer B (0.05/19.95/80.00 (*v*/*v*/*v*) TFA/water/acetonitrile) to 0% buffer A: 100% and buffer B in 60 min at a flow rate of 8 mL/min. The masses of purified products were verified by MALDI-TOF MS (matrix-assisted laser dissociation ionised-time of flight mass spectrometry) (Voyager DE, Perspective Biosystem, Foster City, CA, USA) in positive detection mode using CHCA (α-cyano-4-hydroxycinnamic acid) as the matrix.

### 4.4. Physicochemical Properties Analyses, Secondary Structure Predictions and Determinations

The physicochemical properties of t-DPH1 and its analogues were determined via the Heliquest (http://heliquest.ipmc.cnrs.fr/cgi-bin/ComputParamsV2.py, assessed on 5 November 2021), and helical wheel plots were constructed to predict their secondary structures [51]. Then, circular dichroism (CD) analyses were used to determine the secondary structure of synthesised peptides, which was carried out on a JASCO J815 Spectropolarimeter (JASCO Inc., Easton, MD, USA). The peptide samples (100 µM) were prepared in 10 mM NH_4_Ac solution and 50% TFE/NH_4_Ac (*v*/*v*), respectively, and were measured in the range of 190–250 nm. K2D3 (http://cbdm-01.zdv.uni-mainz.de/~andrade/k2d3/, accessed on 5 November 2021), the online analysis web server, was used to analyse the collected results.

### 4.5. MIC and MBC Determinations

To assess the antimicrobial activity of t-DPH1 and its designed analogues, MIC and MBC assays were conducted. Seven microorganism strains were used in the assay: Gram-positive bacteria *S. aureus* (ATCC 6538), *E. faecalis* (NCTC 12697) and *MRSA* (NCTC 12493); Gram-negative bacteria *E. coli* (ATCC 8739), *P.* *aeruginosa* (ATCC 9027) and *K. pneumoniae* (ATCC 43816); yeast, *C. albicans* (ATCC 10231).

For MIC assay, the microorganisms were initially incubated in Mueller-Hinton broth (MHB) (for bacteria) or yeast extract peptone dextrose broth (YPD-B) (for yeast) overnight and sub-cultured to achieve their respective logarithmic growth phases (5 × 10^5^ CFU/mL). Then, 99 μL of each microorganism and 1 μL of the tested peptide solution were incubated in a 96-well plate. The final on-plate peptide concentrations were from 1 to 512 μM. Except for the peptide group, four other groups were set when conducting this experiment, including a negative control group (PBS), a vehicle control group (1% DMSO), a positive control group (bacteria: 20 μg/mL norfloxacin; yeast: amphotericin B) and a blank control group (MHB or YPD-B with no microorganism culture). Thus, the MIC value represents the lowest concentration of peptides at which no visible growth of the microorganism after 24 h incubation [52]. For the MBC assay, 10 μL of the medium from each clear well was inoculated onto a Mueller–Hinton agar (MHA) (for bacteria) or yeast extract peptone dextrose agar (YPD-A) (for yeast) plate and incubated for 24 h for measurement MBC values.

### 4.6. MBIC and MBEC Determinations

To assess the antibiofilm activity of t-DPH1 and its designed analogues, the MBIC and MBEC assays were conducted. Bacteria types mentioned in Section 4.5. were chosen to be tested in this assay. The bacteria were cultured in the Tryptic Soy Broth (TSB, for Gram-positive bacteria) or Lysogeny broth (LB, for Gram-negative bacteria). The final concentration of peptide solution was from 1–256 μM. Bacteria culture without any treatment served as growth control and sterile PBS served as negative control. For the determination of MBIC, cultures at 10^6^ CFU/mL were incubated with the tested peptide in a 96-well plate (100 μL/well) for 24 h. After incubation, phosphate buffer saline (PBS) was used to wash every well. Then, the biofilm was fixed by 125 μL of methanol (90%, *v*/*v*) for 10 min, and the wells were dried and stained with 125 μL of crystal violet (0.1%, *w*/*v*) for 30 min, and then excess stain was removed. After drying, stained biofilm in each well was dissolved using 150 μL of acetic acid (30%, *v*/*v*). After dissolving, the glacial acetic acid was transferred to a new 96-well plate, and the absorbance at 595 nm was determined by the Synergy HT plate reader (Biotech, Minneapolis, MN, USA). MBIC was the minimum concentration of the peptide that displayed no biofilm formation.

For the MBEC assay, the bacteria were cultured in the 96-well plate for 24 h to form the mature biofilm. Then, the bacterial culture was removed, and the biofilm was further washed using PBS. Then, the fresh broth that contained different peptide concentrations (1–256 μM) was added to the plate for 24 h incubation at 37 °C. The subsequent steps were consistent with the MBIC assay, as mentioned above.

### 4.7. Sytox-Green Bacteria Cell Membrane Permeabilisation

SYTOX Green Nucleic Acid Stain (Life Technologies, Cramlington, UK) can quickly penetrate cells with compromised plasma membranes but does not cross the membranes of living cells, making it a useful indicator of dead cells.

*S. aureus* (ATCC 6538) were cultured in TSB, and *E. coli* (ATCC 8739) were cultured in LB at 37 °C overnight and then were subcultured to reach the logarithmic growth phase. Then, the growth medium was centrifuged at 1000× *g* for 10 min at 4 °C to collect the bacterial cells, where after, 5% TSB or 5% LB in 0.85% NaCl solution were used to wash the cells twice. Next, the pellet was resuspended. Next, the peptide solutions at the concentration of respective MIC and 2 × MIC were added in a black 96-well plate and incubated with bacteria cells at 37 °C for two hours. After incubation, the cells were stained with 5 μM SYTOX^TM^ green nucleic acid stain and incubated for 5 min in the dark at 37 °C. The fluorescent intensity was measured with a Synergy HT plate reader (Biotech, Minneapolis, MN, USA) by an excitation and emission wavelength of 485 and 528 nm, respectively. Bacteria cells treated with 5% TSB or LB medium served as negative control; bacteria cells treated with 70% (*v*/*v*) isopropanol for one hour served as positive control; 5% TSB or LB medium without bacteria cells served as blank control.

### 4.8. Time–Killing Kinetics Determination

The kinetic time–killing assay was conducted to compare the killing rate of t-DPH1 and its analogues against *S. aureus* (ATCC 6538) and *E. coli* (ATCC8739). The bacteria culture was prepared the same way as for the MIC and MBC assay (mentioned in Section 2.6). First, a suspension culture (10^6^ CFU/mL) of bacteria was mixed with peptide solutions at the concentrations of respective MIC and two × MIC in sterile 1.5-mL tubes. Then, the aliquots were removed from culture tubes at 0, 5, 10, 15, 30, 60, 90, 120 and 180 min intervals. The bacteria at different time points were seeded onto MHA plates and incubated at 37 °C for 24 h before colony counting. The bacteria cultured without any treatment was employed as the growth control and bacteria culture treated with PBS served as negative control.

### 4.9. Resistance Induction by Serial Passages

Based on the results of MIC assay, synthesised peptides t-DPH1, t-DPH1-K4, t-DPH1-5K and t-DPH1-6K showed excellent ability against Gram-negative *E. coli*. Therefore, *E. coli* was selected to test whether the MIC value changed after consecutive passages. The drug resistance induction assay was conducted based on the reported methods [53]. Briefly, MIC values of t-DPH1, t-DPH1-K4, t-DPH1-5K and t-DPH1-6K against *E. coli* (ATCC 8739) were determined and recorded. After incubation, the bacterial cells growing at 1/2 MIC values were harvested and inoculated into fresh MHB for another MIC assay. After 20 h incubation, cells growing at 1/2 MIC from the previous passage were harvested and assayed for the MIC. The process was repeated for 12 cycles, and MIC values of each cycle were recorded.

### 4.10. Assessment of Mammalian Cell Proliferation Inhibitory Effect

The MTT assay was conducted to verify the proliferation inhibitory effect of t-DPH1 and its analogues. Human cancer cell lines (NCI-H838 (ATCC^®^ CRL-5844, human non-small cell lung cancer)), (NCI-H157(ATCC^®^ CRL-5802, human non-small cell lung cancer cell line)), (U251MG (ECACC 09063001, human neuronal glioblastoma cancer cell line)), (PC-3 (ATCC^®^ CRL-1435TM, human prostate carcinoma cancer cell line)) and normal human cell lines (HMEC-1(ATCC^®^-CRL-3243, human microvascular endothelial cell line)) and (HaCaT (ATCC^®^-PCS-200-011, human keratinocyte cell)) were purchased from the American Type Culture Collection (ATCC, Manassas, VA, USA) and the European Collection of Cell Cultures (ECACC, Sallisbury, UK) for this assay. For the MTT assay, 8000 cells/per well were seeded in the 96-well plate for 24 h. Subsequently, experiment groups were dosed with fresh serum-free medium containing different concentrations of peptides (100 μM to 10 nM) in 3 replicates. Negative control groups and positive control groups were dosed with the fresh serum-free medium containing equal amounts of PBS, and 0.1% (*w*/*v*) Triton X-100, respectively. Then the 96-well plate was incubated at 37 °C with 5% CO_2_ for 24 h. After, ten microliters of MTT solution (5 mg/mL) (Sigma-Aldrich UK Ltd., Gillingham, UK) were added to each well and incubated in a dark environment for two hours. Then, the solution in each well was removed and 100 μL of DMSO were added and then the plate was shaken for 10 min on a shaking incubator before detecting the OD value by use of a Synergy HT plate reader (BioTek, Minneapolis, MN, USA) at λ = 570 nm.

### 4.11. Haemolysis Assays

The haemolysis assay was performed by mixing 2% (*v*/*v*) fresh defibrinated horse blood (TCS Biosciences Ltd., Buckingham, UK) with peptides in different concentrations. Before the test, horse blood was rinsed with PBS three to four times until the supernatant was clear. Next, peptide solutions (from 100 μM to 1 nM) were incubated with the suspension of red blood cells at 37 °C for two hours. The blood cells treated with 1% Triton-X 100 were used as the positive control, and the blood cells treated with PBS were used as the negative control. After incubation, 100 μL of the supernatant from each sample were transferred to a new 96-well plate, and the OD value of each well was measured with a Synergy HT plate reader (BioTek, Minneapolis, MN, USA) with the absorbance set to 570 nm.

### 4.12. Statistical Analyses

All the results were obtained from at least three replicates of experiments. Data were analysed using GraphPad Prism 8.0 software (GraphPad Software Inc., San Diego, CA, USA). Data are shown as the mean values +/− SD. The *p*-value was calculated by multiple *t*-test and one-way ANOVA test from the mean values of the indicated data. Significant differences are demonstrated with asterisks (* *p* < 0.05; ** *p* < 0.01; *** *p* < 0.001; **** *p* < 0.0001).

## 5. Conclusions

In conclusion, we discovered a novel peptide, t-DPH1, with broad-spectrum antimicrobial activity and antiproliferative activity. A series of cationicity-enhanced analogues of t-DPH1 were designed, and t-DPH1-K4 is expected to become a drug candidate with dual effects due to the fact of its enhanced antibacterial and anticancer activities, low haemolytic activity and cytotoxicity. Through serial passages of *E. coli*, t-DPH-K did not evoke any bacterial resistance. When modifying natural peptides, increasing the net charge is an effective approach to improve the bioactivity, but it is necessary to pay attention to the charge threshold of peptides and balance the charge quantities and other characteristics like hydrophobicity and amphipathicity.

## Figures and Tables

**Table 1:** Sequences and physiochemical properties of t-DPH1 and its analogues predicted by Heliquest online website.

Peptide Name	Peptide Sequence	Net Charge (z)	Hydrophobicity (H)	Hydrophobic Moment (μH)
t-DPH1	GLWSKIKNVAAAAGKAALGAL-NH_2_	4	0.425	0.337
t-DPH1-K4	GLWKKIKNVAAAAGKAALGAL-NH_2_	5	0.380	0.414
t-DPH1-5K	GLWKKIKNVAKAAGKAALGAL-NH_2_	6	0.318	0.452
t-DPH1-6K	GLWKKIKNVAKAAGKAAKGAL-NH_2_	7	0.190	0.513
t-DPH1-6KW	WLWKKIKNVAKAAGKAAKGAL-NH_2_	7	0.297	0.443

**Table 2:** The proportion of α-helix of t-DPH1 and its analogues.

Peptide Name	% of α-Helix in 50% TFE
t-DPH1	53.64
t-DPH1-K4	69.33
t-DPH1-5K	63.02
t-DPH1-6K	60.38
t-DPH1-6KW	54.42

**Table 3:** MICs/MBCs (μM) of t-DPH1 and its analogues against different microorganisms.

	t-DPH1	t-DPH1-K4	t-DPH1-5K	t-DPH1-6K	t-DPH1-6KW
*S. aureus* (ATCC 6538)	8/16	2/4	2/2	4/8	128/128
Methicillin-resistant *Staphylococcus aureus* (MRSA) (NCTC 12493)	16/32	2/4	2/4	32/64	128/256
*Enterococcus faecalis* (*E. faecalis*) (NCTC 12697)	128/256	32/32	8/16	256/512	512/>512
*E. coli* (ATCC 8739)	2/4	1/2	2/4	2/4	2/4
*K. pneumoniae* (ATCC 43816)	8/16	2/4	2/4	4/8	64/64
*P. aeruginosa* (ATCC 9027)	16/32	8/16	4/8	8/16	64/128
*C. albicans* (ATCC 10231)	64/128	32/64	256/512	64/128	512/>512

**Table 4:** MBICs/MBECs (μM) of t-DPH1 and its analogues against different microorganisms.

	t-DPH1	t-DPH1-K4	t-DPH1-5K	t-DPH1-6K	t-DPH1-6KW
*S. aureus* (ATCC 6538)	16/>256	8/256	4/>256	16/>256	128/>256
MRSA (NCTC 12493)	32/>256	8/>256	4/>256	64/>256	>256/>256
*E. faecalis* (NCTC 12697)	256/>256	64/>256	16/>256	>256/>256	>256/>256
*E. coli* (ATCC 8739)	8/256	2/128	4/64	2/256	4/256
*K. pneumoniae* (ATCC 43816)	16/256	4/128	4/64	8/256	64/>256
*P. aeruginosa* (ATCC 9027)	32/>256	16/256	8/128	16/>256	128/>256

**Table 5:** IC_50_s (uM) of t-DPH1 and its analogues against tested cell lines.

	PC-3	H838	H157	U251MG	HMEC-1	HaCaT
t-DPH1	14.67	23.51	10.20	34.25	29.85	175.6
t-DPH1-K4	18.99	10.11	6.143	32.18	46.02	63.37
t-DPH1-5K	2.605	1.796	1.546	2.679	3.897	3.332
t-DPH1-6K	55.65	186.1	88.28	593.7	340.2	151.4
t-DPH1-6KW	118.4	180.8	427.3	1499	293.2	119.3

**Table 6:** HC_50_ values of t-DPH1 and its analogues against horse red blood cells.

Peptides	HC_50_ (μM)
t-DPH1	106.4
t-DPH1-K4	444.8
t-DPH1-5K	19.39
t-DPH1-6K	834.6
t-DPH1-6KW	2094

## Data Availability

The t-DPH1 biosynthetic precursor-encoding cDNA is deposited in the NCBI Database under accession number: OK631446.

## Supplementary Materials

The following are available online at https://www.mdpi.com/article/10.3390/antibiotics10121529/s1, Figure S1: The reverse-phase HPLC chromatograms of (**A**) t-DPH1, (**B**) t-DPH1-K4, (**C**) t-DPH1-5K, (**D**) t-DPH1-6K and (**E**) t-DPH1-6KW. The gradient of buffer B (0.05/19.95/80.00 (*v*/*v*/*v*) TFA/water/acetonitrile) is displayed by the solid blue line. The arrow in each chromatogram indicates the elution peak of the peptide; Figure S2: The mass spectra of (**A**) t-DPH1, (**B**) t-DPH1-K4, (**C**) t-DPH1-5K, (**D**) t-DPH1-6K and (**E**) t-DPH1-6KW were obtained by MALDI-TOF. The arrows indicate the observed [M+H]^+^ ion peaks, the sodium and potassium ion adducts.
