# Supplementary material for: Exploration of the Structure–Function Relationships of a Novel Frog Skin Secretion-Derived Bioactive Peptide, t-DPH1, through Use of Rational Design, Cationicity Enhancement and In Vitro Studies
Source: Antibiotics (Basel). 2021 Dec 14;10(12):1529. doi: 10.3390/antibiotics10121529 (PMC8698721; doi:10.3390/antibiotics10121529)
Supplement: Supplementary file 1 [file antibiotics-10-01529-s001.zip › antibiotics-1479542-supplementary.pdf]

Exploration of the Structure-function Relationships of a Novel Frog Skin Secretion-derived Bioactive Peptide, t-DPH1, through use of Rational Design, Cationicity Enhancement and in *vitro* Studies

Supplementary Materials

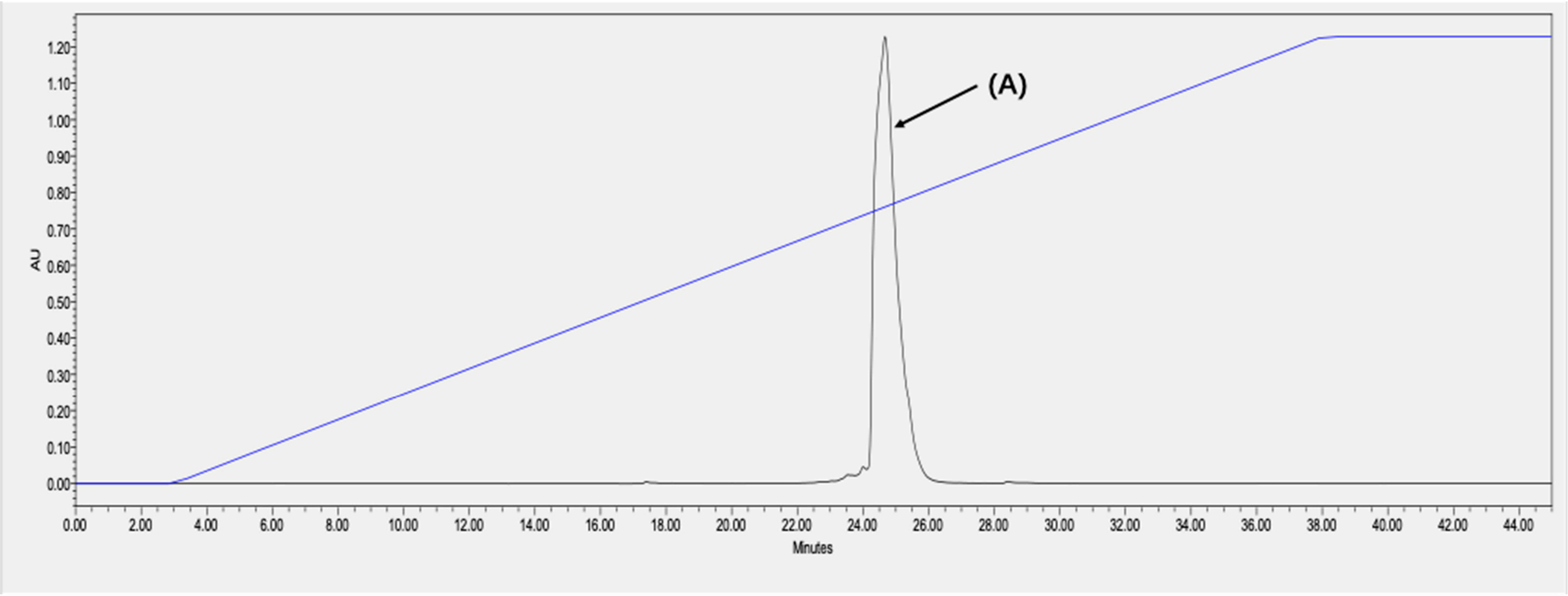

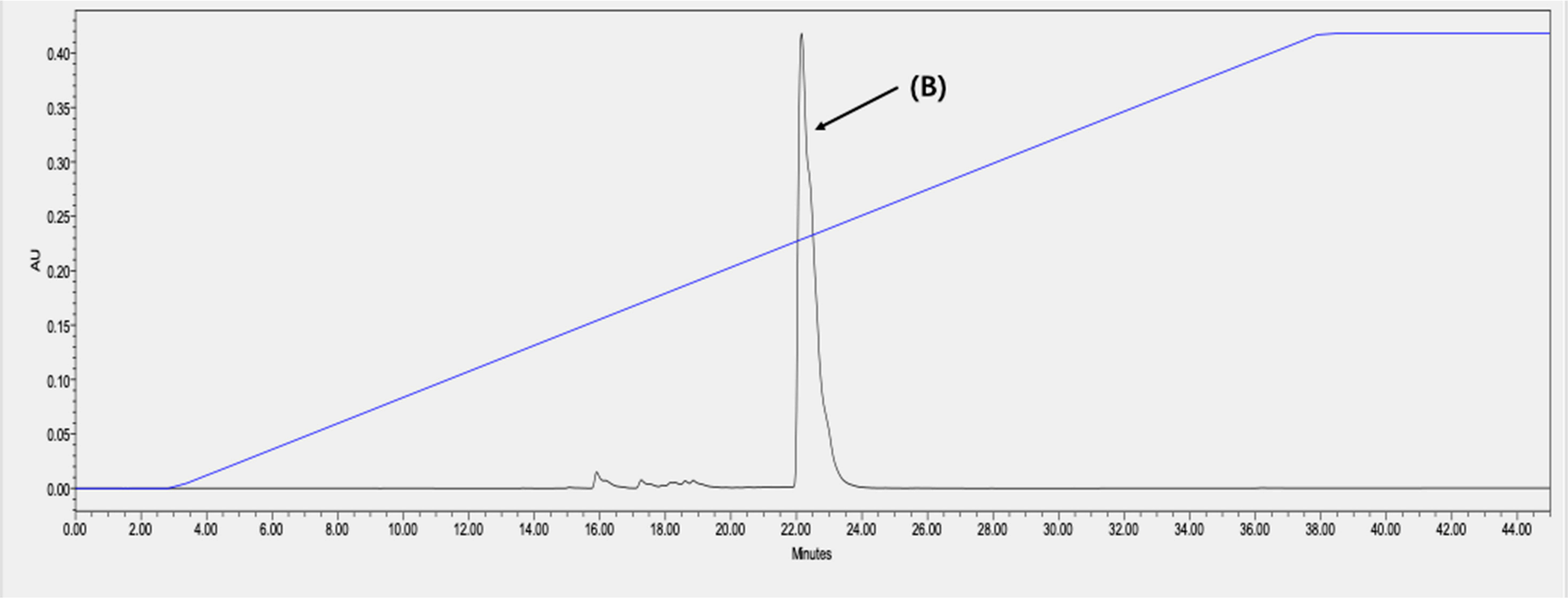
(B)
AU
0.40
0.35
0.30
0.25
0.20
0.15
0.10
0.05
0.00
0.00 2.00 4.00 6.00 8.00 10.00 12.00 14.00 16.00 18.00 20.00 22.00 24.00 26.00 28.00 30.00 32.00 34.00 36.00 38.00 40.00 42.00 44.00
Minutes

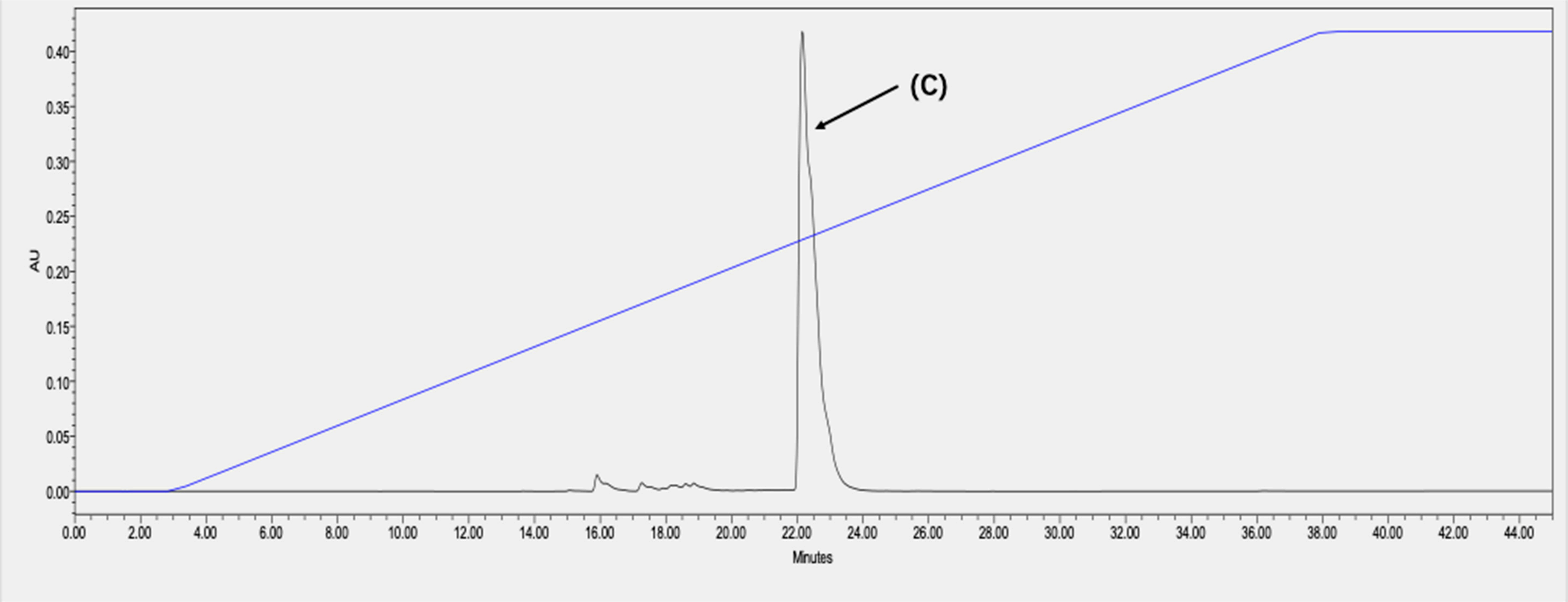
(C)
AU
0.00
0.05
0.10
0.15
0.20
0.25
0.30
0.35
0.40
Minutes
0.00 2.00 4.00 6.00 8.00 10.00 12.00 14.00 16.00 18.00 20.00 22.00 24.00 26.00 28.00 30.00 32.00 34.00 36.00 38.00 40.00 42.00 44.00

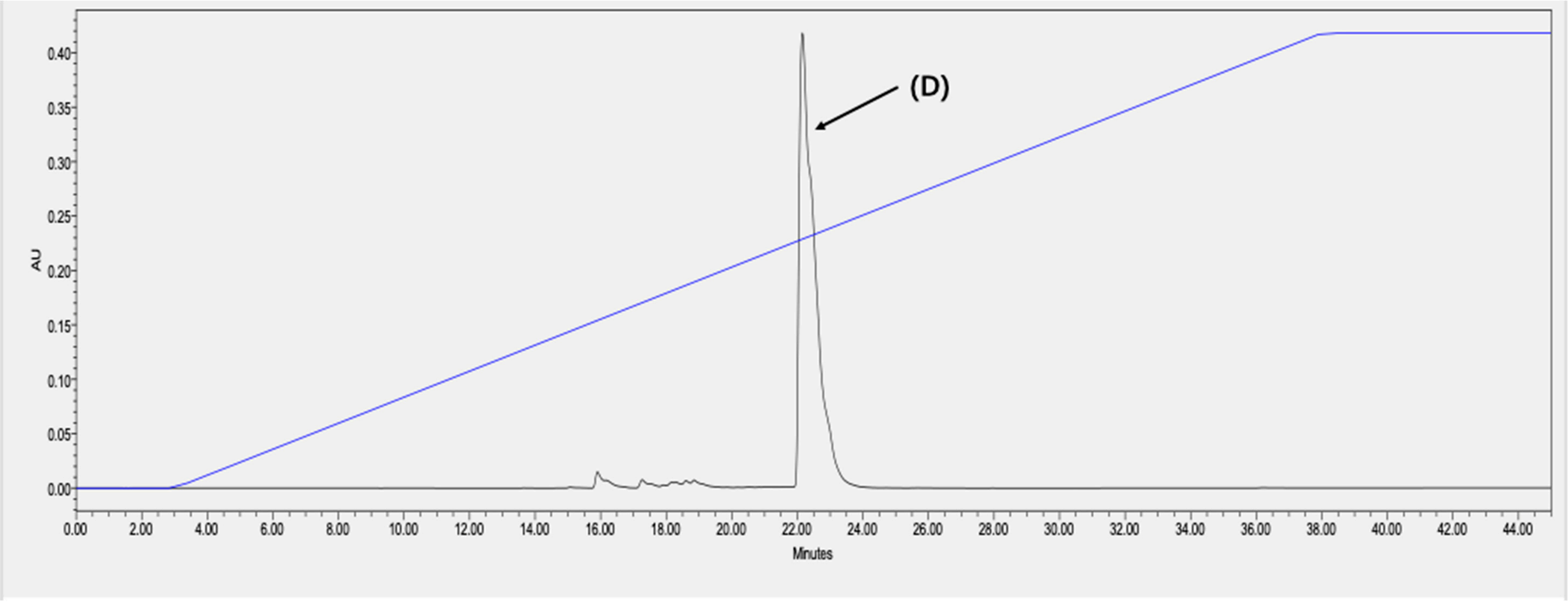
(D)
AU
0.00
0.05
0.10
0.15
0.20
0.25
0.30
0.35
0.40
0.00
2.00
4.00
6.00
8.00
10.00
12.00
14.00
16.00
18.00
20.00
22.00
24.00
26.00
28.00
30.00
32.00
34.00
36.00
38.00
40.00
42.00
44.00
Minutes

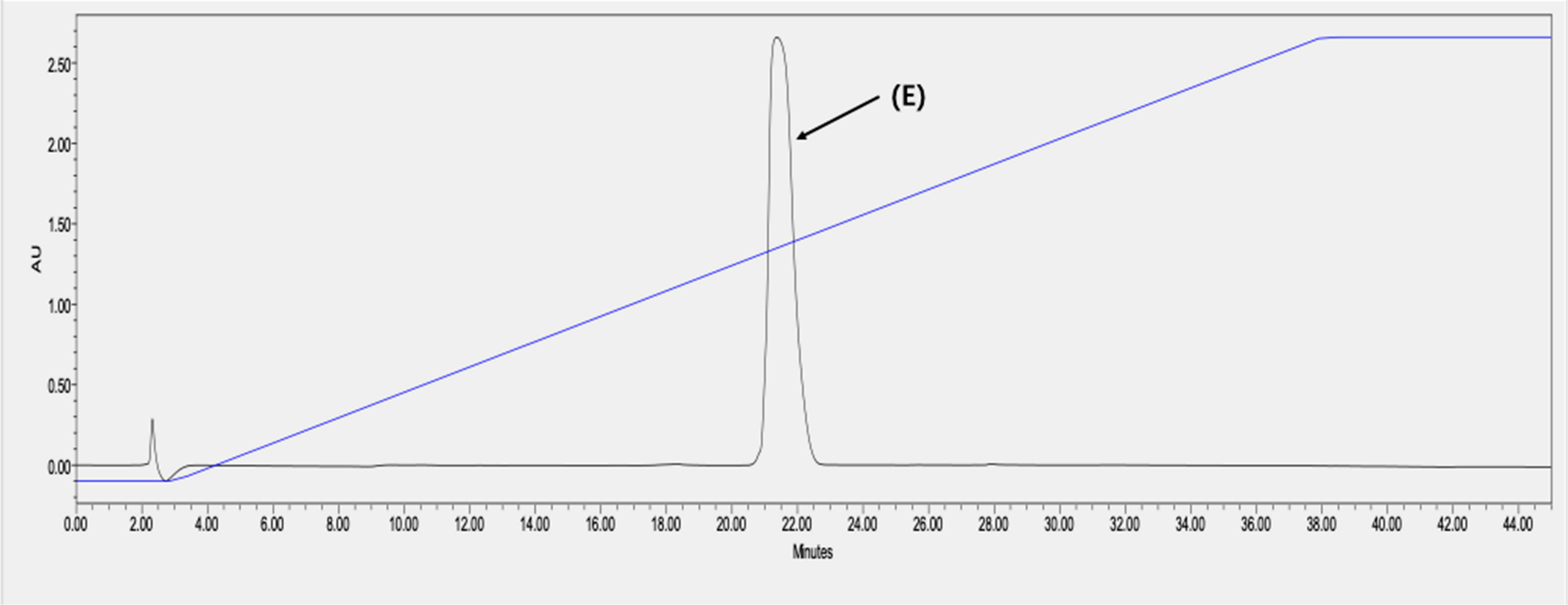

Figure S1. The reverse-phase HPLC chromatograms of (A)t-DPH1, (B) t-DPH1-K4, (C) t-DPH1-5K, (D) t-DPH1-6K, (E) t-DPH1-6KW. The gradient of buffer B (0.05/19.95/80.00 (v/v/v) TFA/water/acetonitrile) is displayed in a solid blue line. The arrow in each chromatogram indicates the elution peak of the peptide.

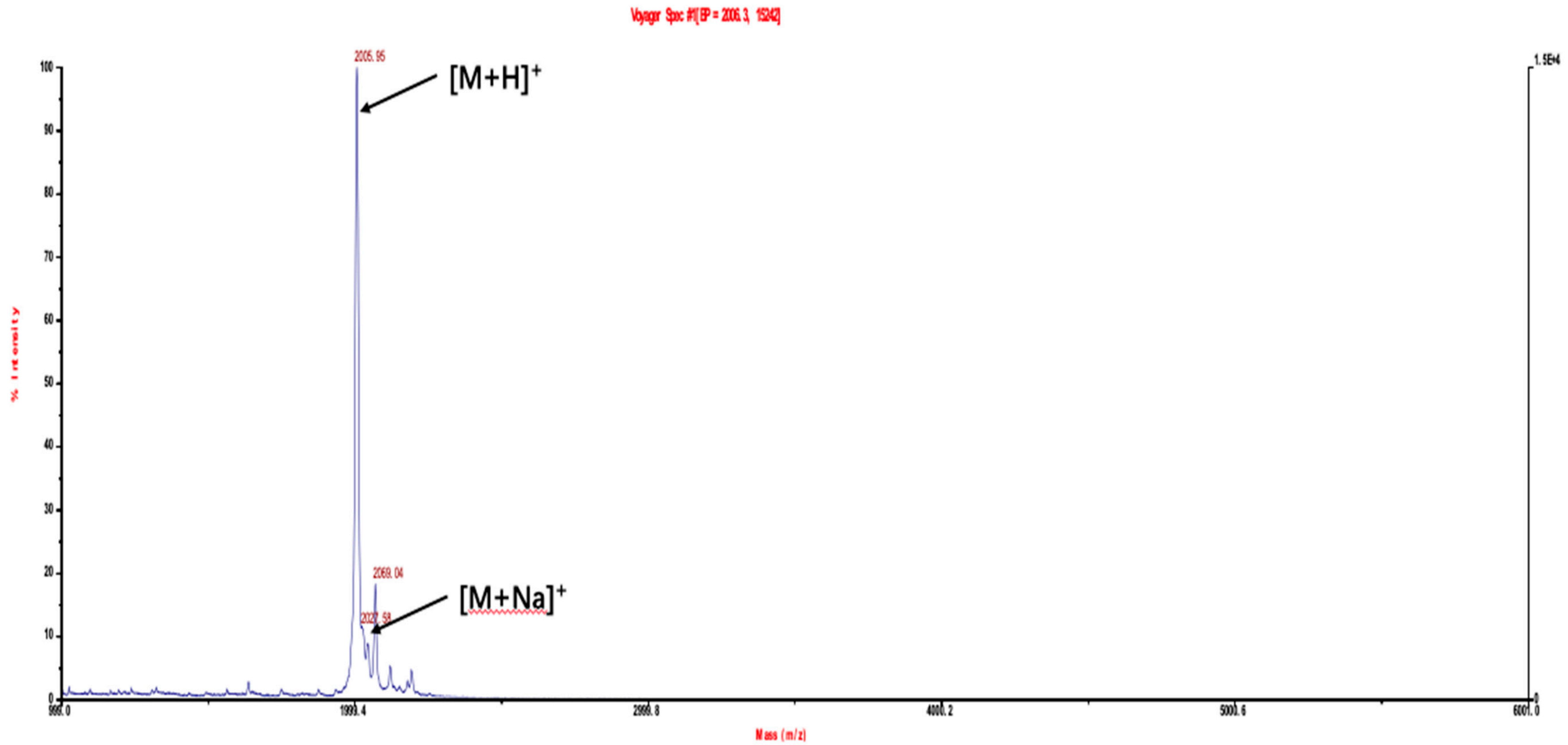
Voyager Spec #1[BP = 2006.3, 15242]
[M+H]+
[M+Na]+
2005.95
2069.04
2027.58
% Intensity
Mass (m/z)
1.5E+4
999.0
1999.4
2999.8
4000.2
5000.6
6001.0

(A)

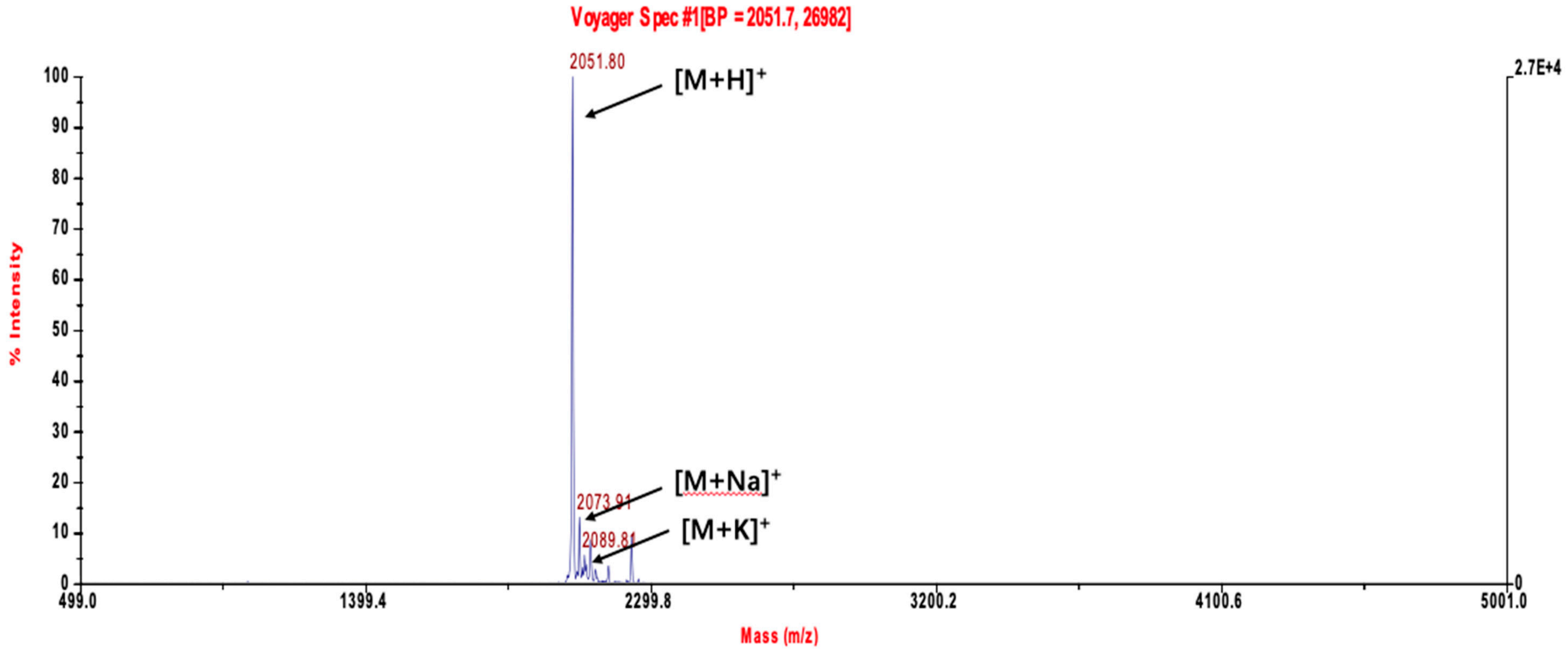

Voyager Spec #1[BP = 2051.7, 26982]
2051.80
[M+H]+
2.7E+4
% Intensity
2073.91
[M+Na]+
2089.81
[M+K]+
Mass (m/z)
499.0
1399.4
2299.8
3200.2
4100.6
5001.0

(B)

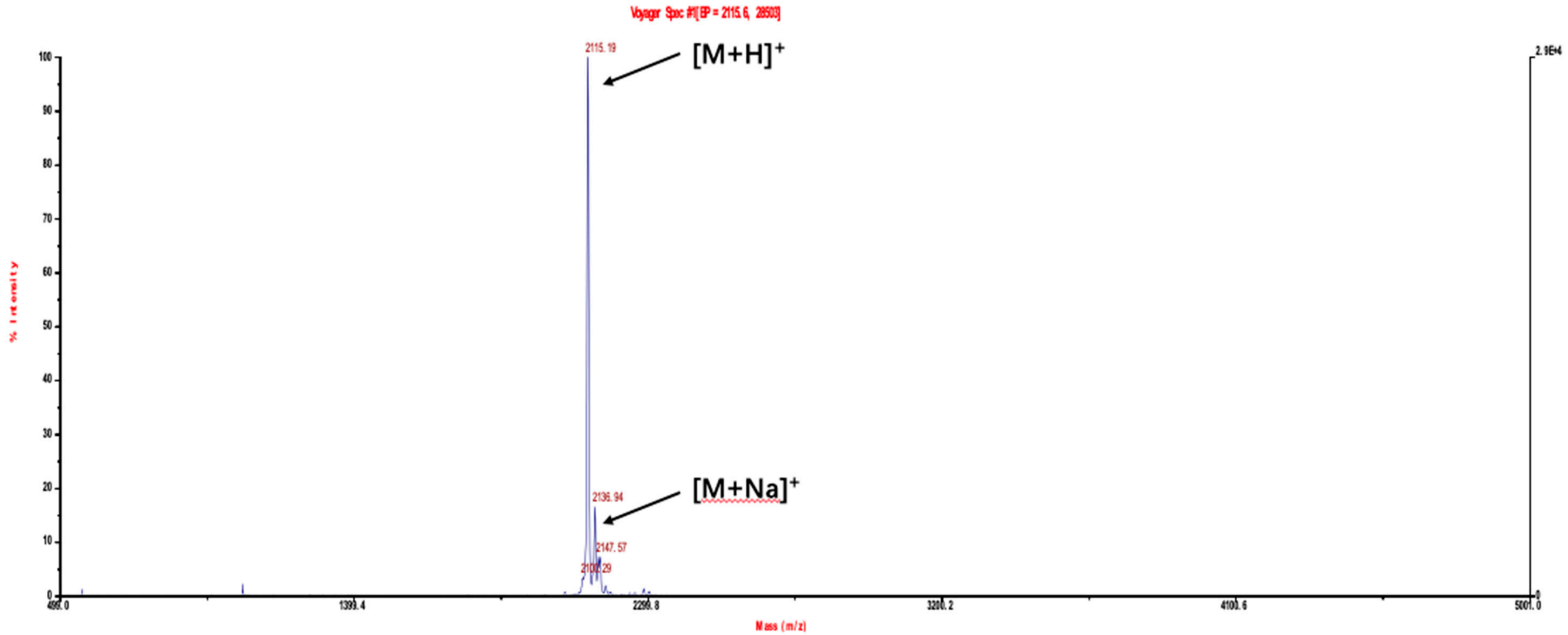
Voyager Spec #1[BP = 2115.6, 28503]
2115.19
[M+H]$^+$
2136.94
[M+Na]$^+$
2147.57
% Intensity
Mass (m/z)
1399.4
2299.8
3200.2
4100.6
5001.0
2.9E+4

(C)

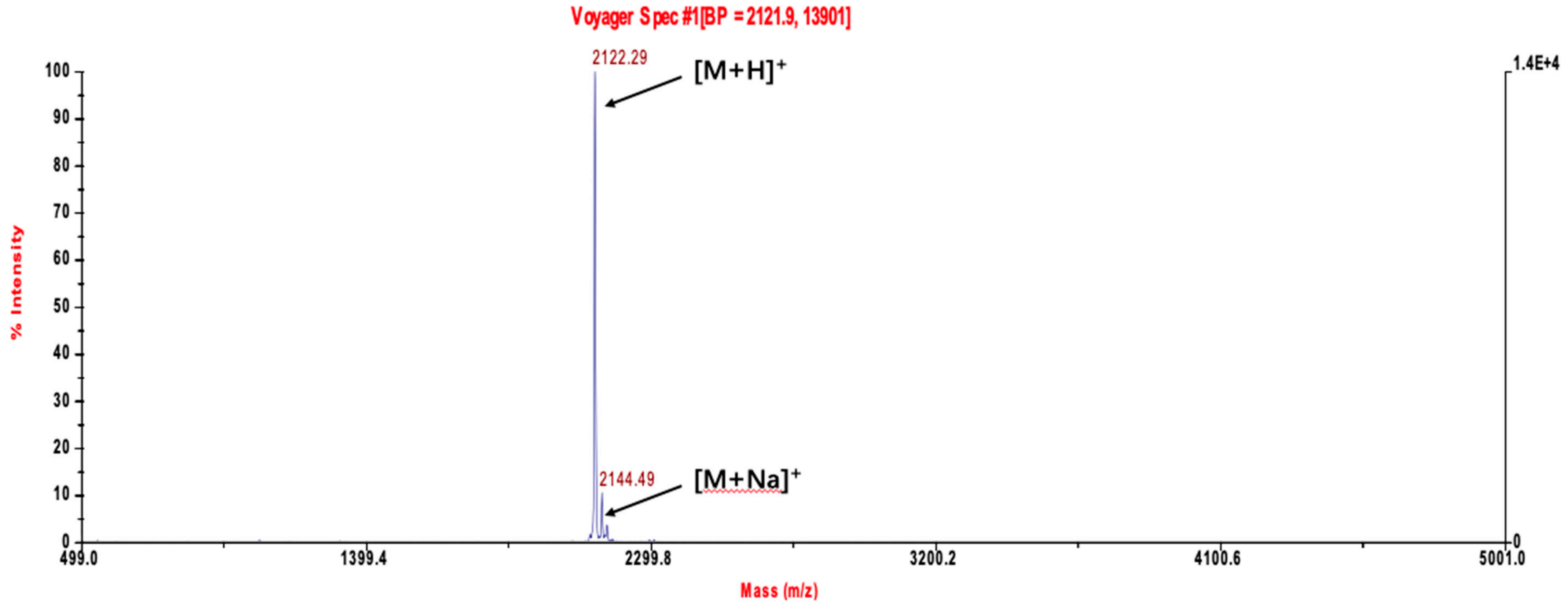

Voyager Spec #1[BP = 2121.9, 13901]
2122.29
[M+H]+
2144.49
[M+Na]+
% Intensity
100
90
80
70
60
50
40
30
20
10
0
1.4E+4
0
499.0
1399.4
2299.8
3200.2
4100.6
5001.0
Mass (m/z)

(D)

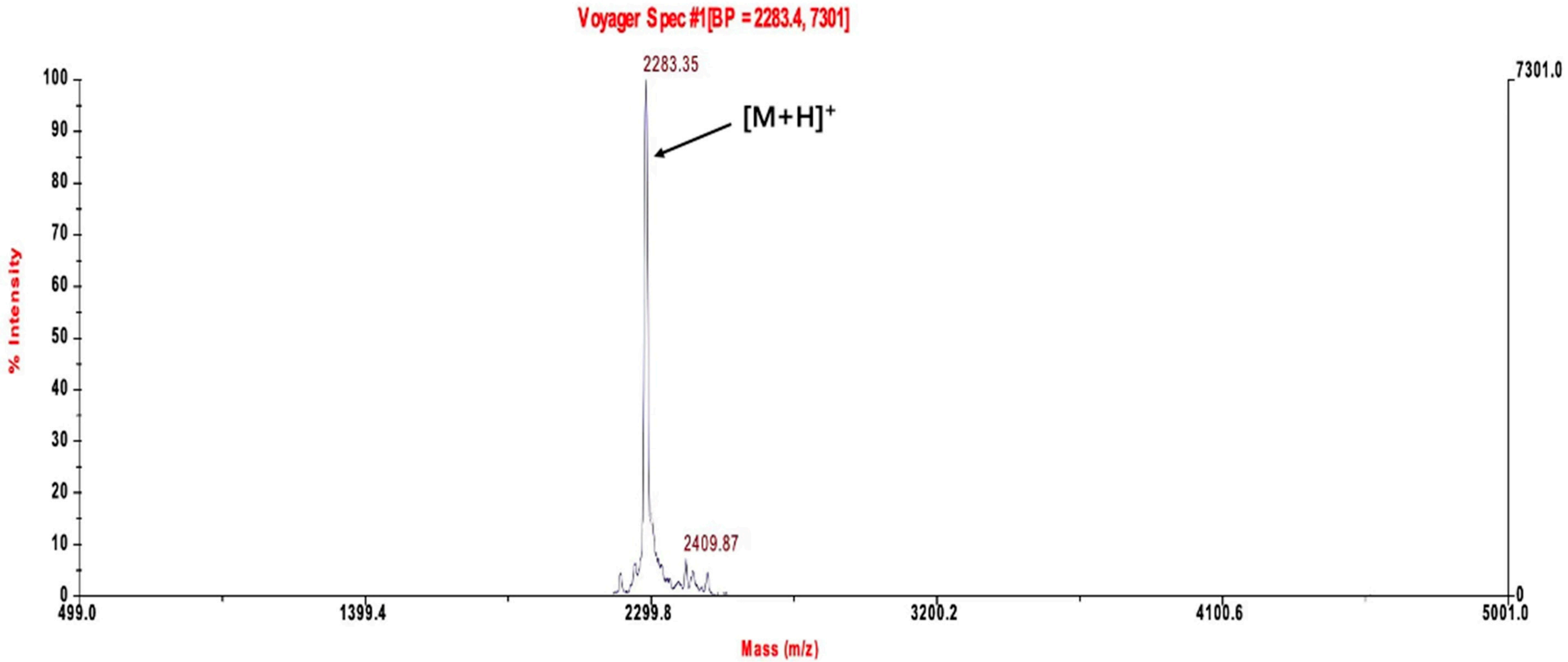

(E)

Figure S2. The mass spectra of (A)t-DPH1, (B) t-DPH1-K4, (C) t-DPH1-5K, (D) t-DPH1-6K, (E) t-DPH1-6KW obtained by MALDI-TOF. The arrows indicate the observed $[M+H]^+$ ion peaks, the sodium and potassium ion adducts.
